# Endovascular and percutaneous embolization of a giant post traumatic arteriovenous fistula of inferior epigastric vessels

**DOI:** 10.1186/s42155-024-00455-5

**Published:** 2024-05-06

**Authors:** Venkata Subbaih Arunachalam, Smily Sharma, Jineesh Valakkada, Anoop Ayyappan, Jayakrishnan Radhakrishnan, Santhosh Kumar Kannath

**Affiliations:** Department of Imaging Sciences and Interventional Radiology, Sree Chitra Tirunal Institute for Medical Sciences and Technology, Trivandrum, India

**Keywords:** Post-traumatic arteriovenous fistula, Large venous sac, Percutaneous embolization

## Abstract

**Background:**

Arteriovenous fistulas involving the anterior abdominal wall can result from trauma. Such fistulas may remain asymptomatic and undetected for a prolonged duration of time. They tend to recruit multiple arterial feeders with remodelling in the feeding arteries, making them challenging to treat.

**Case presentation:**

We discuss a rare case of a 60-year-old male who presented with complaints of a progressive painless swelling in right lower abdomen. There was a history of blunt injury to abdomen at the same site during alleged road traffic accident 3 years ago. On CT angiography, an arteriovenous fistula was localised to the anterior abdominal wall arising predominantly from the right inferior epigastric artery with a giant venous sac and terminating as a tortuous single venous channel into the right external iliac vein. Few other small feeders were also seen arising from branches of right superior epigastric artery along Winslow’s pathway. The main challenge in endovascular management of this patient was embolization of a high flow shunt with a large venous sac and multiple arterial feeders. The dominant arterial feeder was embolized using vascular plug. The superficial location of the lesion offered an additional percutaneous window besides endovascular approach. The venous sac was percutaneously accessed and embolized using n-butyl cyanoacrylate after balloon occlusion of outflow vein. On follow up ultrasonographic evaluation at 3 months, near complete thrombosis of the venous sac was achieved.

**Conclusions:**

Traumatic arteriovenous fistulas involving the inferior epigastric vessels are rare clinical entities. CT angiogram and digital subtraction angiography help in the optimal diagnosis and treatment planning. The use of mechanical embolization devices to cause flow arrest offers an opportunity to use liquid embolic agents which offer better percolation within the lesion. Interventional radiology offers an ideal management of these complex high flow fistulas with a good technical success and acceptable safety profile.

## Background

Post traumatic arteriovenous fistulas in the anterior abdominal wall are very rare. They may have delayed presentation after the initial traumatic insult, making them complex to treat, owing to remodelling in the feeding artery, recruitment of additional arterial feeders and secondary venous hypertension. We present a challenging case of post traumatic arteriovenous fistula (AVF) involving inferior epigastric vessels with a giant venous sac and multiple arterial feeders and discuss the interventional management strategy which resulted in its successful treatment.

## Case presentation

A 60-year-old male with no known comorbidities presented with a gradually increasing painless swelling in right lower quadrant of abdomen for 1 year. There was a history of road traffic accident 3 years back with anterior abdominal wall injury at the same site by steering wheel of his four-wheeler. However, he did not undergo a hospital assessment during that time in view of absence of any debilitating symptoms. On examination, a smoothly marginated non tender pulsatile swelling was seen in right iliac fossa in paramedian location, with an audible bruit on auscultation.

**Fig. 1:**
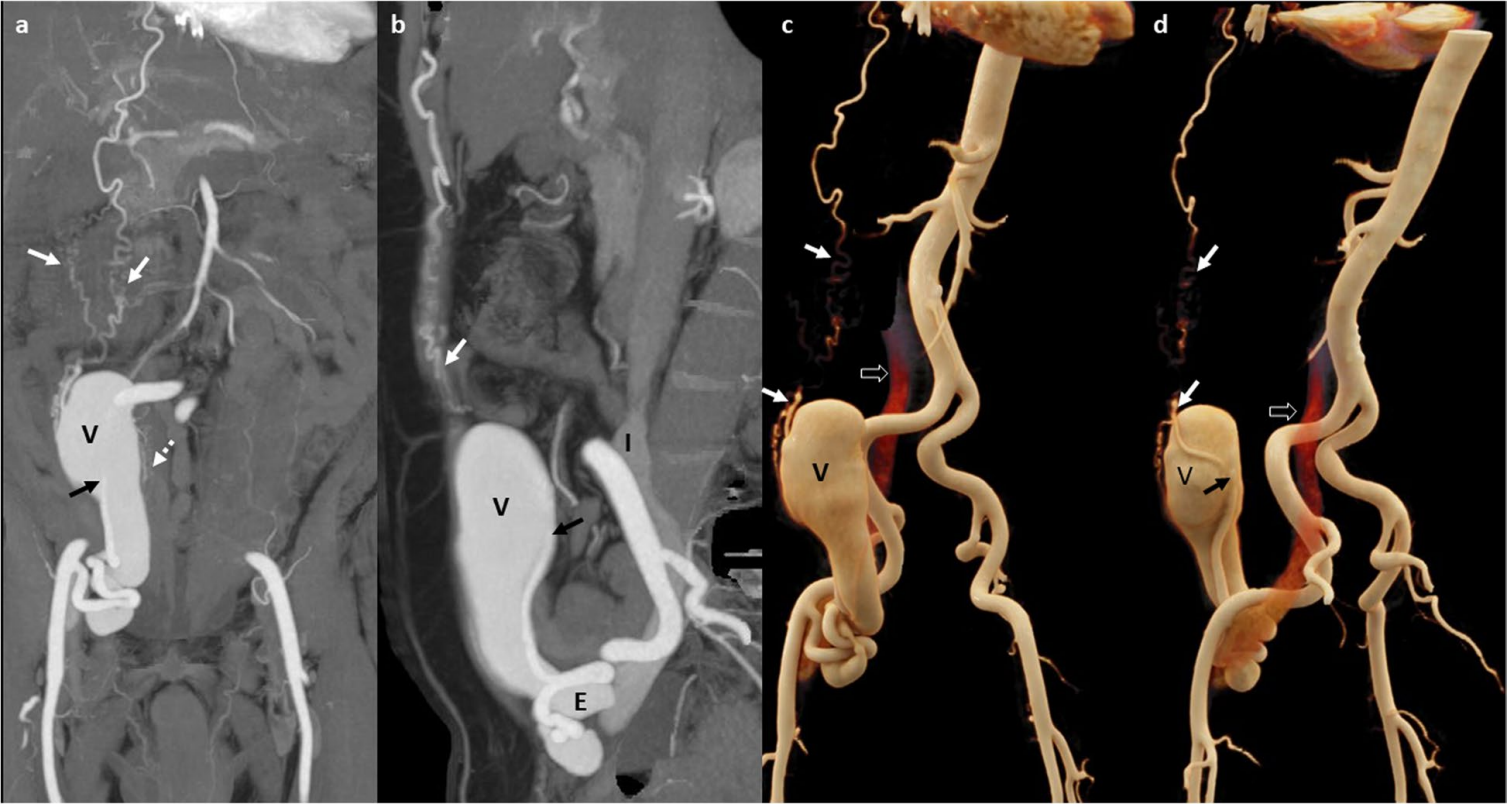
CT angiogram MIP images in coronal (**a**) and sagittal (**b**) planes and cinematic VRT images (**c** anterior and **d **posterior oblique planes) show dilated and tortuous inferior epigastric artery which is communicating (black arrow) with a large venous sac (V) in right lower abdominal wall, posterior to lower third of right rectus abdominis muscle. Multiple other small arterial feeders from superior epigastric artery (white arrow) and inferior epigastric artery (dashed arrow) are also seen. Early arterial opacification of venous sac (V), external iliac vein (E) and IVC (I) is seen (block arrows in **c** & **d**)

CT angiogram showed hypertrophied and tortuous right inferior epigastric artery communicating with an elongated venous sac of size 12 × 4.6 × 4 cm posterior to lower third of right rectus abdominis muscle. Few branches of superior epigastric artery were also seen feeding the venous sac. The venous sac was seen terminating as a tortuous venous channel into the right external iliac vein. Arterial phase opacification of venous sac, right external iliac and common iliac veins was seen, suggestive of AVF (Fig. 1).

Embolization of the right inferior epigastric arterial feeder of the AVF was attempted. The feeding artery was accessed after cross over from a left femoral arterial access (6Fr). Angiogram confirmed fistulous communication between right inferior epigastric artery and vein with a grossly enlarged venous sac in right iliac fossa. Flow related hypertrophic changes were seen in the feeding inferior epigastric artery.

**Fig. 2:**
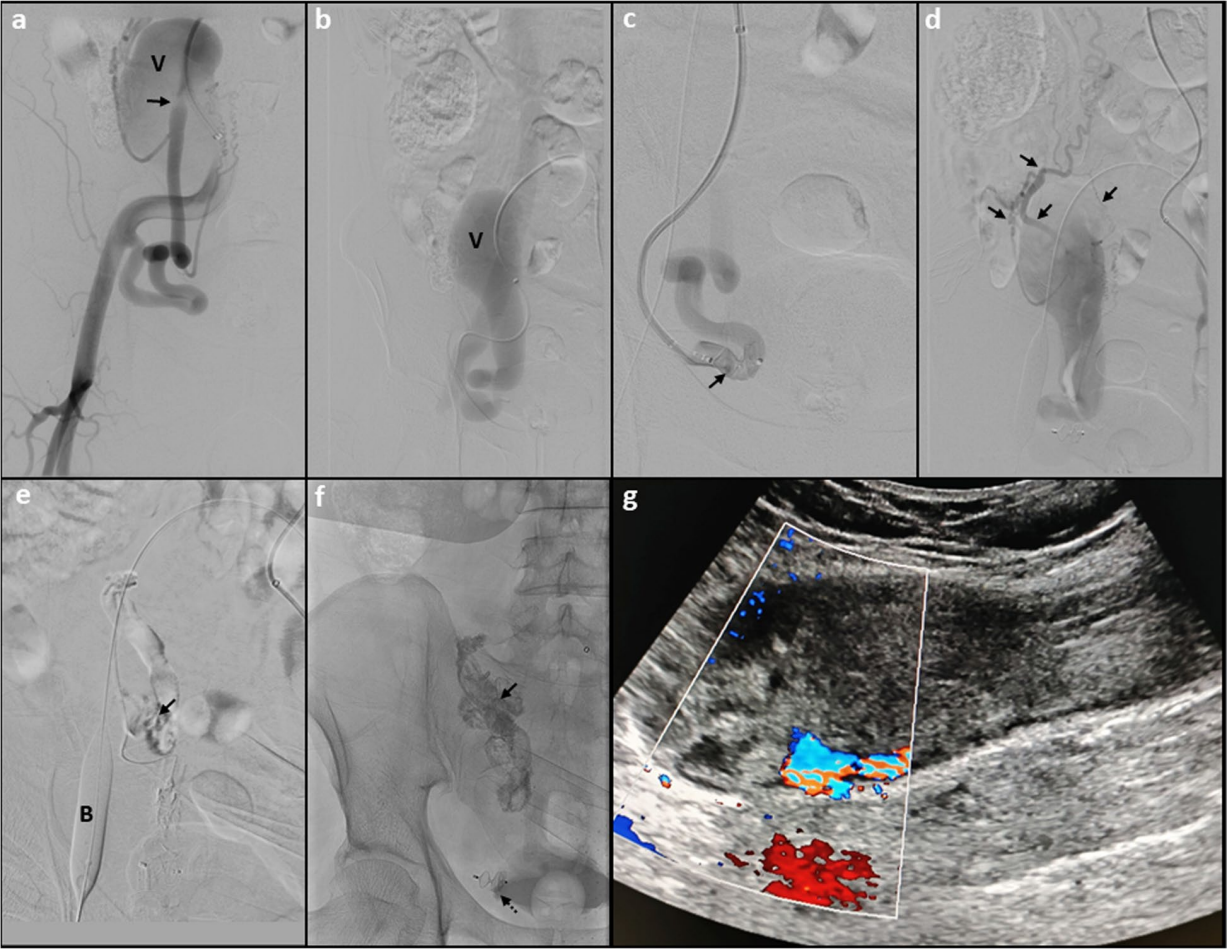
Images showing management of AV fistula using endovascular and percutaneous embolization with follow up: **a** Digital subtraction angiogram image shows tortuous and dilated right inferior epigastric artery having a fistulous communication (arrow) with a large venous sac (labelled as “V”) in right lower abdomen. **b** The venous sac (V) is seen draining into right external iliac vein, with further opacification of external common iliac vein and IVC. **c** Deployment of an Amplatzer vascular plug II (arrow) is seen to cause reduction in antegrade flow. **d** Angiogram images following cannulation of right internal thoracic artery show residual filling of the venous sac via smaller arterial feeders from superior epigastric artery (arrows). **e** Image showing percutaneous embolization of the venous sac with n-butyl cyanoacrylate liquid embolic agent (arrow) following flow arrest using arterial plug embolization and transient balloon (B) occlusion of outflow vein. **f** Spot fluoroscopic post embolization image shows the amplatzer vascular plug in-situ (dashed arrow) with n-butyl cyanoacrylate cast within the venous sac (solid arrow). **g** Sagittal ultrasound image of the right lower anterior abdominal wall taken 3 months after the procedure showing near complete thrombosis of the venous sac with minimal residual colour uptake seen along the posterior aspect of the sac

The inferior epigastric arterial feeder was cannulated and angiogram repeated using a 6Fr Envoy guide catheter. Flow arrest into the fistulous communication was achieved using an Amplatzer Vascular Plug (AVP2) at the arterial end with augmentation of stasis by deployment of a 10 mm x 40 mm PTA balloon catheter (Mustang, Boston Scientific) across the venous drainage site. Persistent colour uptake was seen within the venous sac on transabdominal ultrasonography. The right internal thoracic artery was cannulated using a 5Fr Judkin’s Right catheter. The angiogram showed residual filling of the venous sac with arterial feeders from the right superior epigastric artery across collaterals along Winslow’s pathway. The venous sac was accessed via percutaneous approach under ultrasonographic guidance using a 21G micropuncture needle and 3 F short dilator of micropuncture set secured. Contrast venograms were taken before and after balloon inflation across the footplate of the draining vein. After assuring effective balloon occlusion, the sac was embolised using 10mL of 40% mixture of n-butyl cyanoacrylate liquid embolic agent with lipiodol (Fig. 2).

Check ultrasound revealed obliteration of approximately 85% of the venous sac. The post embolization period was uneventful. On follow up at 3 months, the patient was doing well with mild reduction in the size of the palpable lump, without any local site complaints. Ultrasound examination showed thrombosis of approximately 90% of the venous sac with minimal residual colour uptake along the posterolateral aspect, likely from right superior epigastric artery collateral. Now the patient is on regular 3 monthly follow up with a plan of another sitting of percutaneous sclerotherapy with n-butyl cyanoacrylate if there is expansion in the size of non-thrombosed component or the patient experiences any new attributable symptoms.

## Discussion

AVFs are characterized by abnormal communication between arteries and veins. They can be either spontaneous (primary/ congenital AVFs) or secondary to iatrogenic or traumatic insults. The usual sites of abdominal AVFs include kidneys (secondary to trauma or renal biopsy) and uterus (secondary to miscarriage or dilatation and curettage). Post traumatic abdominal AVFs are mainly caused by penetrating injury, accounting for 20% cases. Blunt trauma leads to only 1% of the AVFs [1, 2]. Post traumatic AVFs involving inferior epigastric arteries are very rare, with only a handful of cases reported in literature [3, 4].

Ours was an extremely rare case of an AVF involving inferior epigastric vessels secondary to blunt trauma abdomen 3 years ago. Similar delayed presentations of traumatic abdominal AVFs have been reported in literature, with a retrospective review reporting an interim period of 7–43 years [1, 5]. These long-standing fistulas are often associated with arterial remodelling and recruitment of multiple arterial feeders, as in our case. Secondary chronic venous hypertension can occur with a risk of ischemia and abdominal varices. High flow AVFs also pose a danger of hemodynamic complications like congestive cardiac failure secondary to significant arteriovenous shunting [1, 6, 7]. The large size of the venous sac and superficial location of AVF in our case posed an additional risk of torrential bleeding.

The treatment options for abdominal AVFs include surgical ligation or endovascular embolization. Few of the previously reported small caliber AVFs have also shown spontaneous closure [3]. Surgery is often associated with higher morbidity and may be difficult in long standing fistulas with surrounding adhesions and desmoplasia, making endovascular management the preferred treatment modality [1, 6].

We used endovascular embolization to treat the complex AVF. The dominant feeding inferior epigastric artery was embolised with Amplatzer vascular plug II. The large diameter of the feeding artery precluded the use of coils, although we made an initial attempt but could not achieve a stable coil framework. Vascular plugs have been successfully used for embolization of abdominal AVFs in previously reported cases [8, 9].

The main challenge was residual flow in the venous sac with small and tortuous superior epigastric artery feeders. In view of multiplicity, small size and extensive tortuousity of these collaterals, trans-collateral embolization could not be attempted. Therefore, we decided to percutaneously embolize the venous sac using n-butyl cyanoacrylate liquid embolic agent. Balloon occlusion at the ostium of draining vein helped to achieve stable embolization and prevented the inadvertent distal embolization of the liquid embolic agent beyond the venous sac. Other embolizing agents like Onyx were not feasible due to requirement of large amounts for embolizing the giant venous sac and drawbacks like skin pigmentation. There were no significant post procedural complications and > 90% thrombosis of the venous sac was seen on 3 month follow up ultrasound.

## Conclusion

Traumatic AVFs involving the inferior epigastric vessels are rare clinical entities, with limited reported cases. CT angiogram helps in the optimal diagnosis and treatment planning. Digital subtraction angiography is the gold standard, with multiple interventional radiology techniques offering potential opportunities to manage these complex high flow fistulas with a good technical success and acceptable safety profile.

## Data Availability

Not applicable.

## Abbreviation

AVF: Arteriovenous Fistula
